# Developmental psychology: Sociocultural contexts and self-regulation

**DOI:** 10.1038/s44271-023-00018-9

**Published:** 2023-09-25

**Authors:** Jennifer A. Bellingtier

**Affiliations:** Communications Psychology, https://www.nature.com/commspsychol

**Keywords:** Psychology, Development studies

## Abstract

How do sociocultural differences in the home and school contexts of immigrant children influence their self-regulation? A recent study in *Child Development* suggests the answer may depend on how you measure it.

**Figure:**
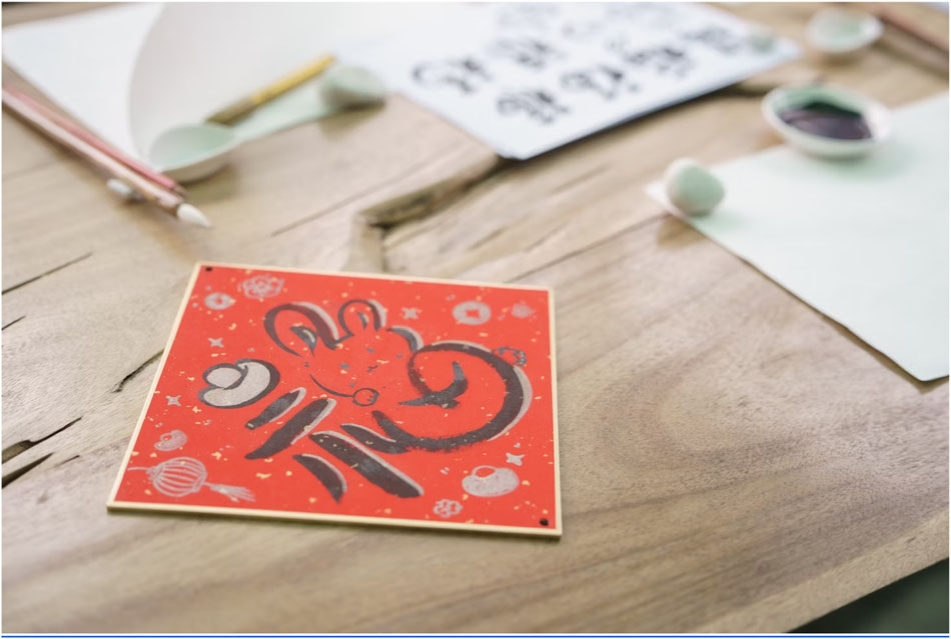
Remi Chow on Unsplash

Self-regulation skills, which predict academic and social adaptation, are socially learned in cultural contexts. For children with immigration backgrounds cultural and self-regulation expectations may be different at home versus at school. For these children, the choice of how to measure self-regulation (i.e., via teacher reports, parent reports, or task-based measures) may be particularly influential for understanding how self-regulation develops.

Christopher L. Gys and colleagues at the University of California, Berkeley, used all three approaches to measures self-regulation in children from Chinese American immigrant families when the children were between 6-9 years of age and then approximately 2 years later^1^. When parent and child had a larger gap in their orientation towards their Chinese culture (e.g., language proficiency, celebration of cultural traditions, choice of friends) the parents were more likely to rate their children as lower in self-regulation. However, cultural orientation had no predictive power for teacher-reported or task-based measures of self-regulation. Parent’s reports of their children’s self-regulation were then best able to predict future behavioral adjustment at home and school.

Using a multi-method, longitudinal design, this study suggests that not only should socio-cultural factors be considered for understanding the development of self-regulation, but that these factors should also be considered when deciding how the construct is measured.
